# Intermediates of Hydrogen Peroxide-Assisted Photooxidation of Salicylic Acid: Their Degradation Rates and Ecotoxicological Assessment

**DOI:** 10.3390/ijms26020697

**Published:** 2025-01-15

**Authors:** Alicja Gackowska, Waldemar Studziński, Alexander Shyichuk

**Affiliations:** 1 Faculty of Chemical Technology and Engineering, Bydgoszcz University of Science and Technology, 85-326 Bydgoszcz, Poland; waldemar.studzinski@pbs.edu.pl (W.S.); szyjczuk@pbs.edu.pl (A.S.); 2 Department of Chemistry, Vasyl Stefanyk Precarpathian National University, 76018 Ivano-Frankivsk, Ukraine

**Keywords:** photooxidation, salicylic acid, dihydroxybenzoic acid, pyrocatechol, ecotoxicity

## Abstract

Accelerated photooxidation of salicylic acid (SA) was performed using UV radiation and hydrogen peroxide. HPLC-MS analysis showed that the primary intermediates are 2,5-dihydroxybenzoic acid, 2,3-dihydroxybenzoic acid, pyrocatechol, and phenol. Deeper oxidation leads to low molecular weight aliphatic acids, such as maleic, fumaric, and glyoxylic. The photooxidation of the main intermediates was carried out in the same conditions. The degradation of SA and its main intermediates follows first-order reaction kinetics. In the case of UV irradiation alone, photodegradation of 2,5-dihydroxybenzoic acid is slightly faster (reaction rate constant is 0.007 min^−1^) compared to SA (0.0052 min^−1^). Other products degrade more slowly than SA. Hydrogen peroxide, in concentrations of 1.8–8.8 mM, accelerates the photodegradation of salicylic acid and intermediate products. An ecotoxicological evaluation of SA and the main products was performed using the EPI Suite^TM^ software. The overall persistence (P_OV_) and long-range transport potential (LRTP) of all transformation products were assessed using OECD P_OV_ and the LRTP screening tool. Salicylic acid and its transformation products have low toxicity. Due to their high solubility, these contaminants can travel considerable distances in the aquatic environment. SA and phenol have LRTP values of 156–190 km. Other products can travel shorter distances (less than 100 km).

## 1. Introduction

Salicylic acid (SA) is a low-toxicity contaminant with an annual global production of approximately 180,000 tons. The main applications are pharmaceuticals, dermocosmetics, and food preservatives. In the pharmaceutical industry, SA is used mainly in the synthesis of acetylsalicylic acid (aspirin), which is one of the most consumed drugs [1]. SA is also a forerunner in the synthesis of other drugs, such as 4-aminosalicylic acid, salicylamide, ethenzamide, phenyl salicylate, and bismuth subsalicylate. In skin care products, SA is used as an effective peeling agent with additional bacteriostatic, fungicidal, and keratolytic effects. SA is a typical component in dermocosmetics to treat acne, melasma, seborrhea, freckles, lentigines, dandruff, sun damage, etc. [2,3].

In the food industry, SA has long been used as a preservative for fruits and vegetables. Exogenous SA reduces respiration and ethylene production, thereby inhibiting post-harvest ripening. This prevents softening and discoloration, maintains the concentration of sugars, organic acids, and polyphenols, and reduces damage during cold storage [4,5]. Being a plant hormone, SA activates the resistance of plant tissues to the development of biotrophic pathogens [6,7]. In agriculture, SA is increasingly used to protect crops from stress caused by drought, salinity, and metal ions [8,9,10,11,12,13].

SA enters the environment in various ways. The main sources of SA contamination are industrial wastewater, municipal sewage, and agricultural leaks. Once taken by a patient, aspirin is rapidly metabolized [14]; then, the formed SA is excreted in the urine and enters the sewage. Therefore, hospital sewages often contain increased amounts of SA and its secondary metabolites [15]. Another source is expired aspirin and other SA-derived medications. If disposed of improperly, SA-derived drugs can cause detectable levels of SA in the environment. Once released into the environment, SA can persist for a long time, negatively affecting aquatic animals. In addition, the presence of SA in the water reservoirs generally increases the resistance of algae to stress factors, causing them to grow faster [16].

The removal of SA from wastewater can be carried out by various methods. Coagulation and electrocoagulation provide rather moderate remediation [17,18,19]. The disadvantage of these techniques is that they produce a precipitate enriched in metal hydroxides. In this respect, membrane separation techniques seem to be more advantageous because they do not generate metal ion contamination [20].

A well-known and versatile method of water purification is adsorption. A commonly used non-polar sorbent is activated carbon, which is well-suitable for SA adsorption [21,22]. The highly porous surface of activated carbon contains graphene-like fragments whose π electrons interact with aromatic SA molecules, thus supporting their adsorption [23]. The presence of basic functional groups on the carbon surface may promote the adsorption of SA [24]. Similar carbonaceous adsorbents for SA are biochars obtained from agriculture and food waste material by pyrolysis [25,26]. Adsorption removal of SA can also be performed using inexpensive natural materials. For example, gooseberry husks from agricultural waste were activated with orthophosphoric acid and heat treated at 500 °C to enhance SA adsorption [27]. Zeolite minerals have a polar surface that weakly adsorbs SA, but surface modification with cationic surfactants enhances adsorption [28]. The modifier cetylpyridinium chloride provides higher adsorption capacity compared to aliphatic surfactant. This is probably because the aromatic pyridine ring further increases the adsorption interaction [28]. The disadvantage of adsorption methods is the need to regenerate used adsorbents.

Among the biological methods of SA remediation, microalgae technology is considered a promising alternative to conventional activated sludge treatment [29]. By harnessing solar energy and binding CO_2_, microalgae technology meets the criteria for a sustainable remediation strategy [30,31]. The biomass growth and biodegradation of salicylic acid depend largely on the species and strain of microalgae used [32,33]. The cultivating medium also plays a role, with the most important components probably being nitrates, phosphates, potassium, and cyanocobalamin [32]. The microalgae cultivation technology is currently being developed at a laboratory scale and requires scale-up to be applied in real industry.

Advanced oxidation processes are used to break down SA into smaller molecules [34,35]. An important issue is the selection of oxidation conditions so that SA undergoes mineralization to simple compounds and does not create intermediate products, which can be more toxic than the substrate [36]. Typically, advanced oxidation processes use agents with high oxidation potential, such as hydroxide radicals [37]. The use of AOPs is growing steadily due to their low cost, ease of operation, and high mineralization of recalcitrant organic contaminants. Recent reports describe the degradation of tetracycline [38], herbicides and insecticides [39,40], phenol, and nitrophenol [41,42,43]. The electro-Fenton process includes the in situ generation of H_2_O_2_ and its subsequent catalytic decomposition into hydroxyl radicals [44,45].

The efficiency of SA degradation can be significantly increased by combining oxidizing agents with UV radiation. An example is a deep degradation of SA by UV-activated peroxy disulfate using a biochar photocatalyst [46]. The combination of photocatalytic reaction with ozone provides approximately twice the efficiency of SA degradation compared to the photocatalytic reaction alone [47].

The combination of hydrogen peroxide and UV radiation also ensures effective oxidation of SA [48,49,50]. In this case, hydroxyl radicals are formed by the direct photolysis of hydrogen peroxide under the influence of UV radiation [51,52]. Hydroxyl radicals react easily with SA, resulting mainly in 2,5-dihydroxybenzoic acid (2,5-DHBA) and 2,3-dihydroxybenzoic acid (2,3-DHBA) [53,54,55].

Under the combined action of UV and H_2_O_2_, the oxidation products of SA undergo further photooxidation. The novelty of this work is the comparison of the photodegradation kinetics of SA and the three main oxidation products measured under the same experimental conditions. The accelerating effect of hydrogen peroxide was studied for the main intermediates using different concentrations of H_2_O_2_. The ecotoxicological characteristics of major degradation products were also assessed [54,55].

## 2. Results and Discussion

### 2.1. The Influence of H_2_O_2_ on Photodegradation of Salicylic Acid

**Figure 1:**
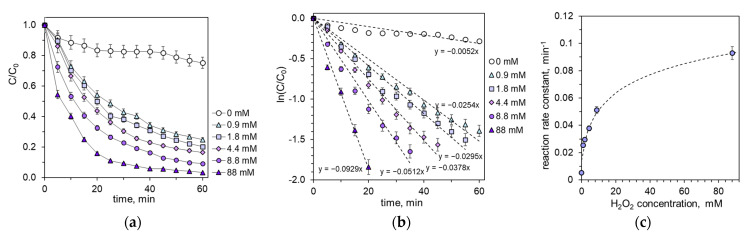
(**a**) Kinetic lines of SA photodegradation depending on the concentration of hydrogen peroxide. (**b**) The kinetic lines transformed according to the kinetic model of a first-order reaction. (**c**) The values of the photodegradation rate are constant depending on the concentration of hydrogen peroxide.

Figure 1a shows the kinetic lines of SA photodegradation. Under the influence of UV irradiation alone, photodegradation occurs at a moderate rate. Approximately 20% of SA degrades for 60 min of irradiation (Figure 1a). Similar results were obtained by Djouder et al. [56], who showed that photodegradation occurs even at low SA concentrations. Photodegradation of SA is further accelerated in the presence of hydrogen peroxide (Figure 1a). The obvious cause is the photolysis of hydrogen peroxide, resulting in aggressive hydroxyl radicals, which attack the SA molecule. Figure 1b indicates that photooxidative degradation follows the first-order kinetic model—the values of the reaction rate constant increase from 0.0052 to 0.0929 min^−1^ (Figure 1b). Figure 1c summarizes the accelerating effect depending on the H_2_O_2_ concentration. At low peroxide concentrations, increasing its amount causes the formation of more hydroxyl radicals and thus increases the rate of SA degradation. However, the accelerating effect of hydrogen peroxide decreases with increasing its concentration (Figure 1c). A similar relationship was also observed in [51]. This dependence on H_2_O_2_ concentration can be explained by the fact that at higher concentrations, hydrogen peroxide can act as a radical scavenger [57,58].

### 2.2. Identification of Salicylic Acid Photodegradation Products

#### Products of SA Photodegradation in the Presence of H_2_O_2_

**Figure 2:**
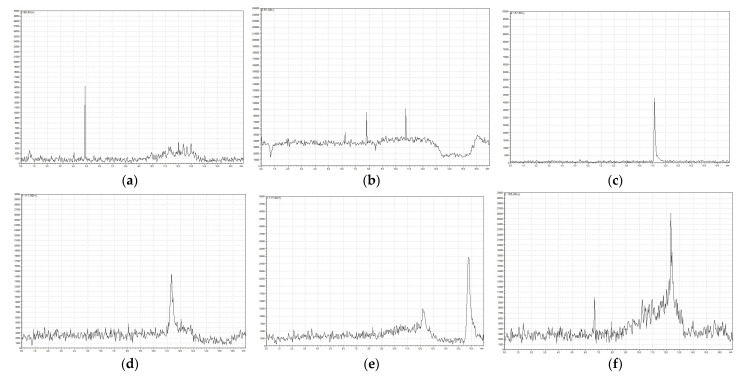
Chromatograms recorded at characteristic *m*/*z* values: (**a**) phenol, (**b**) glyoxylic acid, (**c**) salicylic acid, (**d**) pyrocatechol, (**e**) maleic and fumaric acids, and (**f**) dihydroxybenzoic acids.

Products of SA photodegradation in the presence of hydrogen peroxide were identified using the LC-MS technique. Figure 2 shows exemplary chromatograms of the reaction mixtures, and Table 1 lists the corresponding MS characteristics. Primary products are formed as a result of a direct attack by aggressive hydroxyl radicals, which easily abstract the hydrogen atoms from the aromatic ring of SA. The resulting aromatic radicals bind other hydroxyl radicals, leading to the formation of dihydroxybenzoic acids. The HO• radical is a strong electrophilic reagent. Therefore, the radical attack is directed at positions with increased electron density [53,59]. The dominant formation of 2,5-DHBA is due to the uneven electron density in the aromatic ring of SA. The electron density is higher at position 5, which favors the electrophilic addition of HO• radicals at this position [60].

Under the influence of UV radiation, dihydroxybenzoic acids can lose the carboxyl group, which leads to the formation of dihydroxybenzenes. This study revealed the presence of pyrocatechol (Figure 2d), which is a product of decarboxylation of 2,3-dihydroxybenzoic acid (Table 1). Phenol (Figure 2a) is also present, which is probably formed by the decarboxylation of SA. Similar intermediates were revealed during the oxidation of SA by ozone that was assisted with UV radiation. The main products were found to be 2,5-DHBA, 2,3-DHBA, and pyrocatechol [60,61,62,63].

**Figure 3:**
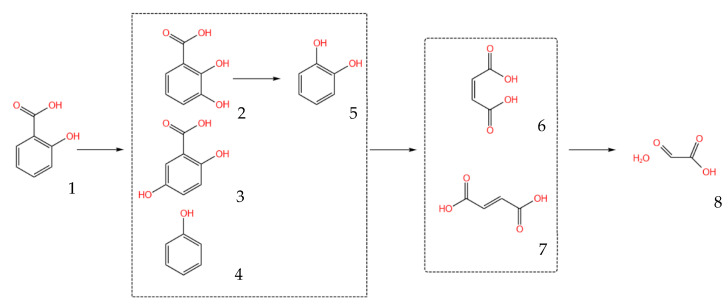
Scheme of transformation of the main products of SA photodegradation: (1) salicylic acid; (2) 2,3-dihydroxybenzoic acid; (3) 2,5-dihydroxybenzoic acid; (4) phenol; (5) pyrocatechol; (6) maleic acid; (7) fumaric acid; and (8) glyoxylic acid monohydrate.

Further radical attacks lead to the opening of the aromatic ring and the formation of aliphatic acids, mainly maleic and fumaric acids (Figure 2e). These unsaturated acids are easily oxidized to low molecular weight glyoxylic acid (Figure 2b) and ultimately to the mineralization product, CO_2_. Based on the identified products, a scheme of salicylic acid transformation under the influence of H_2_O_2_/UV was proposed (Figure 3).

### 2.3. The Influence of H_2_O_2_ on the Degradation of the Main Transformation Products of Salicylic Acid

**Figure 4:**
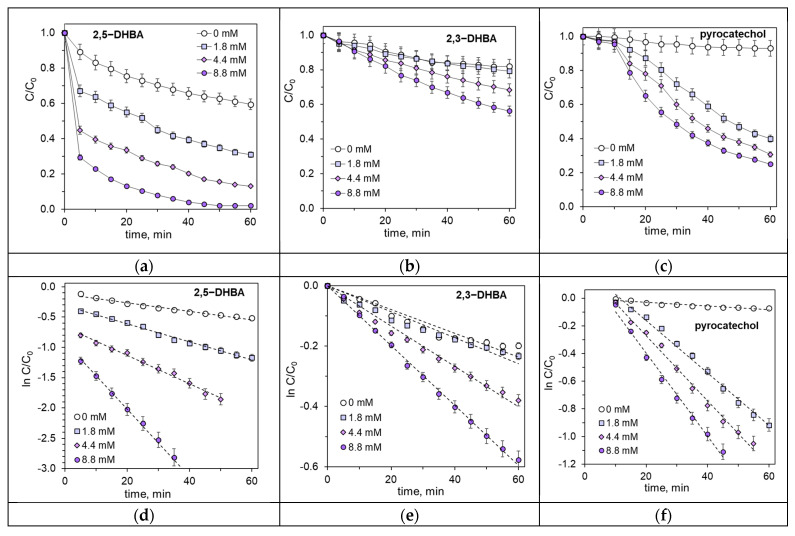
Kinetics of photodegradation of (**a**) 2,5-dihydroxybenzoic acid, (**b**) 2,3-dihydroxybenzoic acid, and (**c**) pyrocatechol depending on the H_2_O_2_ concentration. (**d**–**f**) Transformation of the corresponding kinetic lines (**a**–**c**) on a logarithmic scale.

Similarly to SA, the intermediate products detected in Section 2.2 are reactive molecules and undergo photodegradation. Therefore, their photodegradation kinetics were studied using the same experimental conditions as those used for SA. Figure 4a–c show the kinetic lines of the photodegradation of 2,5-dihydroxybenzoic acid, 2,3-dihydroxybenzoic acid, and pyrocatechol.

Under the influence of UV radiation alone, 2,5-DHBA degrades slightly faster than SA, while 2,3-DHBA degrades slightly more slowly than SA and slower than 2,5-DHBA, too (Figure 4a,b, and Figure 1a). As expected, the addition of H_2_O_2_ to the reaction solution accelerates the photodegradation of both dihydroxybenzoic acids (Figure 4a,b). The degree of acceleration is much greater for 2,5-DHBA than for 2,3-DHBA. Using 8.8 mM H_2_O_2_, 2,5-DHBA was 99.8% decomposed within 60 min (Figure 4a), whereas 2,3-DHBA was only 44% degraded (Figure 4b).

Figure 4c illustrates the photodegradation of pyrocatechol. Without H*_2_*O*_2_*, pyrocatechol decomposes more slowly compared to SA and dihydroxybenzoic acids. Within 60 min of UV irradiation, pyrocatechol degradation is only 7% (Figure 4c). As in the case of dihydroxybenzoic acid, the photodegradation of pyrocatechol is significantly accelerated by H_2_O_2_. Using 8.8 mM H_2_O_2_, pyrocatechol was 75% decomposed within 60 min (Figure 4c). The kinetic lines in Figure 4c also show that the photooxidation of pyrocatechol has an induction period of about 10 min. The obvious explanation is that pyrocatechol acts as a radical scavenger [55].

Figure 4d–f show the kinetic lines transformed on a logarithmic scale. The resulting plots are quite linear, indicating that the hydrogen peroxide-assisted photooxidation of 2,5-DHBA, 2,3-DHBA, and pyrocatechol follows the kinetics of a first-order reaction. The corresponding rate constants are listed in Table 2. The comparison of numerical values in Table 2 confirms the above conclusion: adding H_2_O_2_ accelerates the photodegradation of dihydroxybenzoic acids and significantly accelerates the photodegradation of pyrocatechol. Under the influence of UV radiation enhanced with H_2_O_2_, 2,3-dihydroxybenzoic acid and pyrocatechol decompose more slowly than SA.

### 2.4. Ecotoxicological Evaluation of Salicylic Acid Transformation Products

The identified intermediates make it possible to assess the possible environmental hazard of the SA photooxidation that is assisted by hydrogen peroxide. Table 3 shows the physicochemical properties and environmental characteristics of the photooxidation products calculated using EPI Suite^TM^ 4.11 software.

The boiling point (BP) and vapor pressure (VP) provide information on whether a compound will be emitted into the atmosphere relatively quickly after being released into the environment. Typically, an organic compound is considered volatile if it has 15 or fewer carbon atoms, its vapor pressure is greater than 10 Pa at 25 °C, and its boiling point at atmospheric pressure is less than 260 °C [64]. Table 3 shows that only phenol and pyrocatechol meet these criteria. Salicylic acid and other degradation products have a VP below 7.5 × 10^−2^ mmHg and can, therefore, be classified as low-volatile compounds. The parameters BP and VP indicate that salicylic acid and its transformation products do not tend to evaporate and remain in the gas phase.

The parameter of water solubility (WS) suggests the fate of substances in water bodies. The solubility of salicylic acid in water at 25 °C is 3808 mg L^−1^. The photooxidation products with several –COOH and –OH groups are more soluble in water than SA (Table 3). The good solubility additionally reduces evaporation into the air phase. One can conclude that SA and its photooxidation products do not tend to migrate from water to air. However, the high values of WS suggest that these pollutants can migrate with water over considerable distances. The highly soluble compounds can also be easily absorbed by plants and animals.

Due to its good solubility, salicylic acid is often detected in the aquatic environment. According to Yang et al., SA is one of the three most frequently detected pharmaceutical and personal care products in surface waters [65]. Recorded SA concentrations in aquatic environments range from 0.1 to 16.9 μg L^−1^ in Asia and from 2.8 to 27.8 μg L^−1^ in North America. The concentration of SA in wastewater from the pharmaceutical industry can reach 500 mg L^−1^ [29,66,67]. Benzoic acid and a wide range of benzene derivatives and related compounds are widely used as antibacterial and antifungal preservatives and as flavoring agents in food, cosmetics, hygiene, and pharmaceutical products. Due to their widespread production and use, these compounds are found in the environment, mainly in water, but also in soil and air [68]. One of the main pollutants of this group is 2,4-dihydroxybenzoic acid [63]. Phenolic compounds are also common surface water pollutants. More than 60 different phenols were identified in the aquatic environment in concentrations from 0.065 to 179,000,000 ng L^−1^. The highest concentrations were recorded in surface water channels in India [69].

The log K_OW_ parameter also confirms the hydrophilic nature of salicylic acid and its transformation products. The discussed compounds are characterized by log Kow values in the range from –0.7 to +2.24 (Table 3). This suggests that they are less likely to accumulate in living organisms, sediments, and soils. The values of a BCF parameter less than 12 indicate a low probability of accumulation and bioconcentration in living organisms. It is worth noting that the oxidation products have even lower BCF values than the substrate (Table 3).

The ability of pollutants to bioconcentrate in living organisms is one of the parameters taken into account in assessing a threat posed by the new environmental pollutants. Due to its low potential for accumulation, the presence of SA in the body of humans or animals is mainly detected in serum and urine [70]. However, it has also been observed that salicylic acid can undergo biotransformation to oxidation products, as well as the formation of conjugates with amino acids. Conjugation of salicyluric acid with glycine was found in pigeons. In chickens, no glycine conjugate was formed, but instead, a doubly conjugated metabolite with ornithine was identified in plasma, feces, kidneys, and liver [71]. In the plant kingdom, salicylic acid is widely distributed and has many physiological effects important for plant survival. SA is an important signaling molecule in induced systemic acquired resistance in tobacco, cucumbers, tomatoes, and other plants [72]. Therefore, 2,4-DHBA and its glycosylated form were found to be accumulated after SA application. 2,4-DHBA acts as a potentially bioactive molecule and is mainly stored conjugated with glucose. When 2,4-DHBA is applied exogenously, tea plants accumulated more 2,4-DHBA than SA and showed induced resistance to Ps. camelliae-sinensis (PCS) infection. These results indicate that glucosylation of 2,4-DHBA positively regulates disease resistance in tea plants [73].

The values of overall persistence (P_OV_) and long-range transport potential (LRTP) of all transformation products were assessed using OECD P_OV_ and the LRTP screening tool. [74]. The input parameters were log K_OW_, log K_OA,_ and log K_AW_, half-lives in air, water, and soil, and molar masses of compounds. By taking into account the different migration paths (water, air, and soil), the resulting diagram contains four regions with a specific priority of environmental risk. Region A corresponds to the high values of both LRTP and P_OV_. Examples of persistent pollutants located in region A include hexachlorocyclohexane, p-cresol, and polychlorinated biphenyls. They are considered pollutants of high priority. Region B corresponds to the high values of LRTP and low values of P_OV_, whereas region C corresponds to the low values of LRTP and high values of P_OV_. Pollutants located in these regions are considered to be of moderate priority. Region D corresponds to the pollutants having low values of both LRTP and P_OV_. They are considered to be of low priority [75].

**Figure 5:**
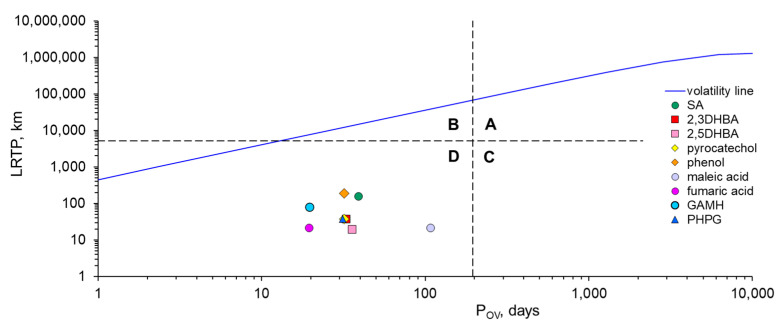
Long-range transport potential (LRTP) vs. overall persistence (P_OV_) of SA and its degradation products. The dashed lines indicate the critical values of LRTP and P_OV_, which are 5097 km and 195 days, respectively. The solid line (labeled “volatility line”) indicates the physical limit of the migration of contaminants in the air.

The LRTP index shows the environmental mobility of the studied products. Salicylic acid and phenol have LRTP values of 156–190 km (Figure 5). The LRTP values decrease with the increase in the number of hydroxyl groups in the molecule. Therefore, other transformation products can be transported at much shorter distances (less than 100 km). The influence of the structure, molar mass, and type of atoms in individual molecules was described by Mostrąg et al. [75]. The overall durability parameter shows similar relationships. The P_OV_ values of the discussed compounds are in the range from 19 to 108 days, with maleic acid being the most stable (Figure 5). Considering the LRTP and Pov values in the framework of classification by Klasmeier et al. [74], salicylic acid and its transformation products can be assigned to the compounds with the lowest priority. The results are similar to the oxidation products ethylhexyl-methoxycinnamate (EHMC) and octyl-dimethyl-para-aminobenzoic acid (ODPABA), which are used as UV filters and are also classified as emerging pollutants [76,77].

**Figure 6:**
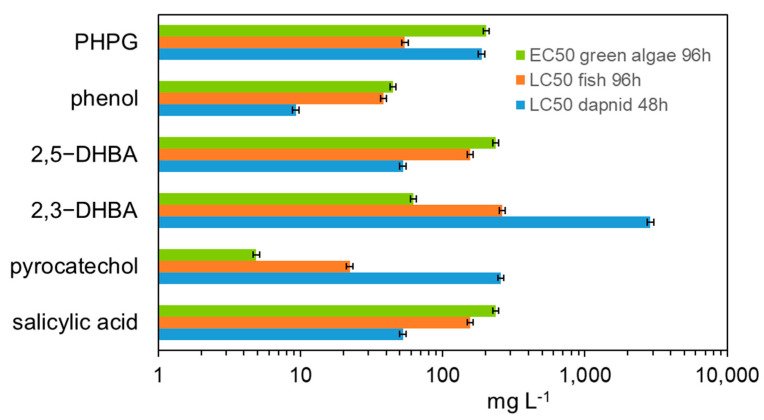
The assessed toxicity of SA degradation products.

The toxicity of salicylic acid transformation products was assessed by determining the acute toxicity for three groups of indicator organisms using the Ecosar module of the Epi Suite program. Such simulations usually provide reliable toxicity results. The obtained results indicate that both salicylic acid and its transformation products have low toxicity. The only toxic product is phenol (Figure 6).

The non-toxic nature of salicylic acid is confirmed by the therapeutic effects of SA on humans, animals, and plants. For example, Mahdavian found that salicylic acid (SA) is a plant hormone that has therapeutic effects against non-biological stresses such as salinity [78]. However, it should be remembered that any contamination may cause undesirable physiological reactions in non-target species. Despite its widespread use, SA can cause acute and chronic toxicity known as salicylism, symptoms of which include nausea, vomiting, dizziness, disorientation, delirium, stupor, psychosis, coma, and even death in the worst cases [79]. Therefore, there is great concern about the presence of SA in municipal and industrial wastewater, and its removal before discharge into the aquatic environment has attracted much attention in recent years [80,81]. In fact, out of 140 emerging contaminants (ECs), SA was recently designated as one of eighteen that should be regulated for wastewater discharge [33,82]. Previous studies have shown that SA-type contaminants are toxic to a wide range of aquatic organisms, causing liver and kidney damage, protein denaturation, and even mucosal bleeding [83,84]. For example, exposure to SA can significantly increase the activity of selenium-dependent glutathione peroxidase and glutathione reductase in the liver of brown trout (*Salmo trutta fario*) and cause nonspecific pathological changes such as fusion of the branchial lobes and epithelial cell hyperplasia [85]. SA also significantly reduces the respiratory capacity of *Mytilus galloprovincialis*, causing neurotoxicity and oxidative stress [86]. Furthermore, SA reduced swimming speed and distance, heart rate, and jaw movements in *Daphnia*, suggesting that SA may act as an ecotoxicological factor affecting both the behavior and physiology of freshwater crustaceans [87]. All these studies emphasize the need for more comprehensive research on the molecular mechanisms underlying SA toxicity in aquatic ecosystems. Kamaya et al. [88] studied the toxicity of benzoic acid and its derivatives with hydroxyl and/or methoxyl groups substituted in the aromatic ring on the freshwater crustacean *Daphnia magna* under neutral conditions (initial pH of 7.45 ± 0.05). Toxicity, expressed as the EC_50_ value, varied largely depending on the number and position of hydroxyl groups. In particular, benzoic acids with ortho-substituted hydroxyl groups were more toxic than benzoic acids with meta- and/or para-substituted hydroxyl groups. Of the compounds tested, 2,4,6-trihydroxybenzoic acid showed the highest toxicity with a 48 h EC_50_ [88]. On this basis, it can be stated that transformation products such as pyrocatechol, 2,3-DHBA, and 2,5-DHBA may also be potentially dangerous to the environment. In addition, hydroxybenzoic acids are precursors of highly toxic quinones [63]. Another toxic product of the photodegradation of salicylic acid is phenol. Phenol can cause serious health problems, such as convulsions, loss of coordination, tremors, respiratory arrest, and muscle weakness. Phenol also causes serious damage to the nervous system. Therefore, phenol is subject to environmental regulations [89].

## 3. Materials and Methods

### 3.1. Reagents

Analytical standards of salicylic acid, 2,5-dihydroxybenzoic acid, 2,3-dihydroxybenzoic acid, and pyrocatechol of 99% purity were purchased from Sigma Aldrich. Hydrogen peroxide (H_2_O_2_) solution (30% *w*/*v*) was purchased from POCh (Gliwice, Poland). The methanol, water, and formic acid used in the chromatographic analyses were purchased from Sigma Aldrich (St. Louis, Missouri, United States).

### 3.2. Experiments on the Photodegradation of Salicylic Acid and Its Transformation Products

Aqueous solutions of salicylic acid, 2,5-dihydroxybenzoic acid, 2,3-dihydroxybenzoic acid, and pyrocatechol had a concentration of 0.36 mM. The photodegradation experiments were carried out using a photoreactor (Heraeus, Germany) equipped with a 150 W medium-pressure mercury lamp (TQ150W) and cooled with tap water to a temperature of 20 ± 1 °C. The photoreactor was placed on a magnetic stirrer to carry out the reaction in the entire volume of the reaction mixture. The polychromatic light used in the photodegradation experiments was characterized by excitation wavelengths of 313, 365, 405, 436, 546, and 578 nm. The corresponding illumination intensity values were 2.5, 5.8, 2.9, 3.6, 4.6, and 4.2 W, respectively.

The concentrations of the tested compounds were determined using a JENWAY 7315 UV–vis spectrophotometer. The measurements were carried out at a wavelength of 300 nm for salicylic acid, 276 nm for pyrocatechol, 324 nm for 2,5-dihydroxybenzoic acid, and 292 nm for 2,3-dihydroxybenzoic acid.

### 3.3. Detection of Salicylic Acid Transformation Products

The transformation products were detected using a Shimadzu UFLC XR liquid chromatograph equipped with an MS detector (LC-MS 2020 Shimadzu) equipped with an electrospray ionization source. A Kinetex^®^ Phenomenex C18 column with dimensions of 3 mm × 100 mm, particle size of 2.6 μm, and pore size of 100 Å was used for the analysis. The mobile phase consisted of water with 0.1% HCOOH (A) and methanol (B). The elution gradient was the following: 95:5 *v*/*v* (time 0); 95:5 *v*/*v* (time 7); 55:45 *v*/*v* (time 10); 35:65 *v*/*v* (time 11); 5:95 *v*/*v* (time 12); 5:95 *v*/*v* (time 14); 95:5 *v*/*v* (time 15); 95:5 *v*/*v* (time 17). The flow rate was 0.4 mL⋅min^−1^, the column temperature was 45 °C, and the injection volume was 5 μL. The DAD detector scanned the spectral range from 220 to 700 nm. The electrospray ionization detector operated using the following parameters: a temperature of the desolvation line at 250 °C, a nebulizing gas flow of 1.5 mL min^−1^, and a heating block temperature of 400 °C. The negative ion with *m*/*z* of 137 was selected to monitor the salicylic acid. In addition, the entire range of 50–750 *m*/*z* was scanned in positive and negative ionization to detect the presence of other analytical signals. The operating conditions of the mass spectrometer were as follows: a drying gas (N₂) flow rate of 1.5 mL⋅min^−1^, gas temperature of 400 °C, nebulizer gas pressure of 30 psi, fragmentor voltage of 70 V a, and capillary voltage of 4000 V.

### 3.4. Determination of Ecotoxicological Characteristics

The physicochemical parameters of SA and its transformation products were determined using the EPI Suite^TM^ 4.11 software. [90]. The software uses quantitative structure–activity relationship (QSAR) models to estimate critical parameters. The following parameters were calculated: melting point (MP), boiling point (BP), water solubility (WS), vapor pressure (VP), bioconcentration factor (BCF), logarithm of octanol/water partition coefficient (Log K_OW_), logarithm of octanol/air partition coefficient (Log K_OA_), logarithm of organic carbon/water partition coefficient (Log K_OC_), logarithm of air/water partition coefficient (Log K_AW_), Henry’s constant (K_H_), LC_50_, and EC_50_. Additionally, the P_OV_ and LRTP parameters were calculated for all SA transformation products using the P_OV_ and LRTP screening tool. [91].

**Figure 7:**
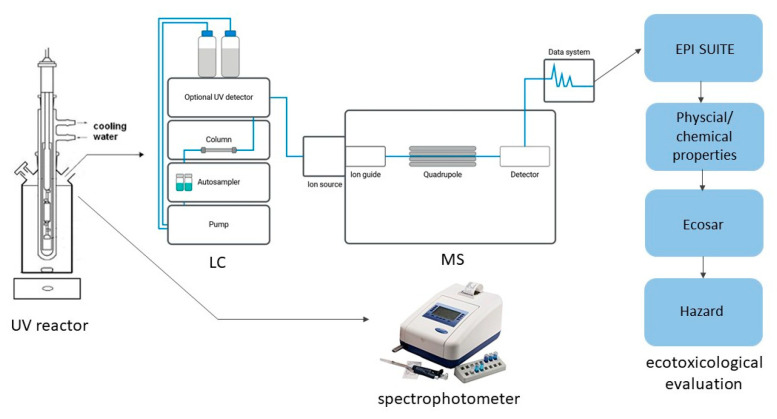
Flowchart of research photodegradation of salicylic acid, product identification, and ecotoxicological assessment.

The overall procedure for the hydrogen peroxide-assisted photodegradation of salicylic acid, product identification, and ecotoxicological assessment is presented schematically in Figure 7.

## 4. Conclusions

The main products of SA photodegradation are 2,5-dihydroxybenzoic acid, 2,3-dihydroxybenzoic acid, pyrocatechol, and phenol. Photodegradation of salicylic acid and its major intermediates follows first-order reaction kinetics. Under the influence of UV radiation alone, 2,5-DHBA degrades faster than SA, while 2,3-DHBA and pyrocatechol degrade more slowly than SA.

The combination of UV radiation with hydrogen peroxide significantly increases the rate of photodegradation. The accelerating effect increases with an increasing H_2_O_2_ concentration. The degree of acceleration is much greater for 2,5-DHBA than for 2,3-DHBA and pyrocatechol. The combined action of UV and H_2_O_2_ is suitable for the decomposition of SA in wastewater from the pharmaceutical industry.

The ecotoxicological parameters of SA and its transformation products were assessed, resulting in a diagram of overall persistence (POV) vs. long-range transport potential (LRTP). SA and its transformation products are located in the D region corresponding to the low values of both LRTP and POV. In other words, SA and its transformation products can be considered as pollutants of low priority.

## Figures and Tables

**Table 1:** Retention times and *m*/*z* values of the main products of SA photodegradation.

Retention Time, T_R_	Compound	Molecular Weight	*m*/*z*
4.87	phenol	94	[M+H]^+^ 95
10.73	glyoxylic acid monohydrate	92.05	[M−H]^−^ 91
11.14	salicylic acid	138.12	[M−H]^−^ 137
11.33	pyrocatechol	110.11	[M+H]^+^ 111
12.12	maleic acid	116.07	[M+H]^+^ 117
12.34	2,3-dihydroxybenzoic acid	154.12	[M+H]^+^ 155
12.43	2,5-dihydroxybenzoic acid	154.12	[M+H]^+^ 155
15.76	fumaric acid	116.07	[M+H]^+^ 117

**Table 2:** Reaction rate constants (min^−1^) of photodegradation of SA and main intermediates depending on H_2_O_2_ concentrations.

Compound	0 mM	1.8 mM	4.4 mM	8.8 mM
salicylic acid	0.0052	0.0295	0.0378	0.0512
2,5-dihydroxybenzoic acid	0.007	0.0148	0.0234	0.0552
2,3-dihydroxybenzoic acid	0.0039	0.0043	0.0067	0.0099
pyrocatechol	0.0013	0.0189	0.023	0.03

**Table 3:** Physicochemical parameters and environmental characteristics of salicylic acid and its photooxidation products: boiling point (BP), melting point (MP), vapor pressure (VP), Henry’s law constant (Henry’s LC), the logarithmic value of n-octanol-water partition coefficient (log K_OW_), logarithmic value of air/water partition coefficient (log K_AW_), logarithmic value of n-octanol/air partition coefficient (log K_OA_), logarithmic value of organic carbon/water partition coefficient (log K_OC_), bioconcentration factor (BCF), and half-life values.

Compound	BP, °C	MP, °C	VP, mmHg	WS, mg/L	Henry’s LC *	log K_OW_	log K_AW_	log K_OA_	Log K_OC_	BCF	Half-Life, h		
											Air	Water	Soil
SA	298	94	8.2 × 10^−5^	3808	1.52 × 10^−9^	2.24	−6.523	8.783	1.573	11.96	19.7	360	720
pyrocatechol	230	46	1.48 × 10^−1^	73,200	5.83 × 10^−1^	0.88	−7.309	8.189	1.746	1.17	2.47	360	720
2.3-DHBA	338	128	2.9 × 10^−7^	26,100	1.48 × 10^−12^	1.2	−10.22	11.96	1.152	1.234	27.6	360	720
2.5-DHBA	338	128	4.36 × 10^−5^	9034	1.48 × 10^−12^	1.74	−10.22	11.96	1.452	2.163	27.6	360	720
maleic acid	285	84	1.26 × 10^−12^	104,000	1.34 × 10^−12^	−0.48	−10.26	10.72	0.41	1.164	29.3	208	416
fumaric acid	285	84	1.26 × 10^−12^	7000	1.34 × 10^−12^	−0.48	−10.26	10.72	0.41	1.164	29.3	208	416
GAMH	249	57	1.04 × 10^−3^	1,000,000	3.13 × 10^−9^	−0.7	−6.894	6.194	−1.054	0.8999	13.8	208	416
phenol	182	40	4.30 × 10^1^	26,200	5.61 × 10^−7^	1.46	−4.866	6.326	1.9	2.419	9.76	360	720
PHPG	289	75	4.16 × 10^−4^	114,000	5.56 × 10^−12^	0.47	−9.643	10.11	0.893	1.091	5.94	360	720

* mol L^−1^ atm^−1.^

## Data Availability

The data presented in this study are available upon request from the corresponding author.
